# Evidence of factors influencing self-medication with antibiotics in LMICs: a systematic scoping review protocol

**DOI:** 10.1186/s13643-018-0758-x

**Published:** 2018-07-21

**Authors:** Neusa Fernanda Torres, Buyisile Chibi, Lyn E. Middleton, Vernon P. Solomon, Tivani Mashamba-Thompson

**Affiliations:** 1 Instituto Superior de Ciências de Saúde - ISCISA (High Institute for Health Sciences), Maputo, Mozambique; 2 Discipline of Public Health Medicine, School of Nursing and Public Health, University of KwaZulu Natal, Durban, South Africa; 3 School of Health Sciences, University of KwaZulu Natal, Durban, South Africa

**Keywords:** Antibiotics self-medication, Factors, Reasons, LMICs

## Abstract

**Background:**

The Sustainable Development Goals (SDGs) emphasize the need for strengthening the capacity of all developing countries in the early warning, risk reduction and management of national as well as global health risks. Despite there being a considerable amount of effort in controlling and promoting the rational use of antibiotics, studies show that the practice of self-medication with antibiotics (SMA) systematically exposes individuals to the risk of antibiotic resistance and other antibiotic side effects. The proposed scoping review aims to map literature on the factors influencing self-medication with antibiotics in low- and middle-income countries (LMICs).

**Methods and analysis:**

The adopted search strategy for this scoping review study will involve electronic databases including PubMed, Web of Knowledge, Science Direct, EBSCOhost (PubMed, CINAHL, MEDLINE), Google Scholar, BioMed Central and World Health Organization library. A two-stage mapping strategy will be conducted. *Stage 1* will screen studies through examining their titles and screening abstracts descriptively by focus and method as stipulated by the inclusion and exclusion criteria. In *stage 2*, the researchers will extract data from the included studies. A parallel screening and data extraction will be undertaken by two reviewers. In accessing the quality of the included studies, the researchers will utilize the mixed methods appraisal tool (MMAT, version 11). The NVivo computer software (version 11) shall be used to classify, sort, arrange and examine relationships in the data, and to extract the relevant outcomes and for the thematic analysis of the studies.

**Discussion:**

The study anticipates finding relevant studies reporting evidence on the factors influencing self-medication with antibiotics in LMICs. The evidence obtained from the included studies will help guide future research. The study findings will be disseminated electronically and in print with presentations being done at relevant platforms, i.e. conferences related to antibiotic use, antimicrobial resistance, health seeking behaviour and the use of medicines.

**Systematic review registration:**

Prospero Registration Number: CRD42017072954

## Background

### Global overview and impact of self-medication with antibiotics

Antibiotic resistance is a global public health problem whereby a sustained proliferation in antibiotic resistance may eventually surpass the rate of new antibiotic development. Studies have consistently documented that the inappropriate and excessive use of antibiotics are the chief contributing factors causing the emergence and selection of resistant bacteria [1–3]. This represents one of the most important worldwide issues for global public health and for patient safety [2, 4, 5]. Regarding the importance of early warning, risk reduction and management of national and global health risks, the Sustainable Development Goals (SDGs) emphasize on the need to strengthen the capacity of all countries with particular mention to developing states [6]. However, factors that may undermine the achievement of that goal include the inappropriate use of antibiotics, increases in health-care service costs, self-medication practices and the consequential development of bacterial resistance as well as side effects [7].

Antibiotic resistance not only impairs the ability to treat common bacterial infections but it is also a major threat to public health, as it increases the health cost with more powerful antibiotics, morbidity and mortality, especially in low- and middle-income countries (LMICs) [8]. The governments of this group of countries are concerned with the indiscriminate use of antibiotics and with the increase in practices of self-medication with antibiotics [9]. At the same time, challenges such as poor health systems, poor supervision and control of antibiotics remain a challenge in LMICs. In addition, poor prescribing and dispenser practices by health workers as well as the failure to comply with guidelines for antibiotics dispensing have been identified in these settings [10]. The World Health Organization (WHO) reports that in LMICs, about 80% of antibiotics are used in the community, of which about 20–50% are used inappropriately [11]. It has also been reported that over 50% of global antibiotic prescriptions are inappropriate, and two thirds of antibiotics available within the pharmaceutical sector are used for self-medication [11].

### Problem statement

Concerted efforts are being made by health authorities in LMICs to consolidate the pharmaceutical sector by strengthening regulation and inspection of drugs [8]. Nevertheless, practices of self-medication with antibiotics are very common and people remain uninformed about the risks of such practices [12]. There is also a notable lack of evidence indicating what factors influence the use of antibiotics for self-medication in LMICs. The existing literature illuminates on the need to understand the phenomena of SMA (self-medication with antibiotics). Furthermore, it also examines the diverse factors facilitating access to antibiotics for self-medication as well as the different sources of such antibiotics [12, 13].

### Significance and aim of the study

This study identifies the need to explore strategies that will aid in controlling, improving and promoting a better use of antibiotics. This is imperative in the development of evidence-based approaches on antibiotic stewardship and management in LMICs. This systematic scoping review aims at mapping literature that evidences factors and practices influencing SMA in LMCs. To achieve the study aim, the specific objectives are the following:

❖ To identify the prevalence of SMA;

❖ To identify factors and reasons influencing SMA;

❖ To identify and describe the sources and types of antibiotics for self-medication;

❖ To identify and describe the health conditions leading to SMA;

❖ To determine the nature and quality of studies reporting evidence on factors influencing for SMA.

The findings from this study will enable the researchers to examine the extent, range and nature of research activity on the factors influencing SMA. The findings will also enable the researchers to identify and describe the dynamic of SMA, as well as the main reasons for it. The information generated will benefit health authorities, health care workers, consumers, pharmacists and public. It will also be useful for educational purposes.

### Methodology

#### Scoping review

The current scoping review protocol was registered and published in the PROSPERO international prospective register for systematic reviews. It is registered under the following registration number: CRD42017072954.The framework adopted for conducting the proposed review is by Arksey and O’Malley [14]. Briefly, the framework involves (i) identifying the research question, (ii) identifying relevant studies, (iii) study selection, (iv) charting the data and (v) collating, summarizing and reporting the results.

#### Identifying the research question

The research question is as follows: What is known from existing literature on factors influencing SMA in LMICs?

The research sub-questions are:

1. Are there evidences on the prevalence of SMA?
2. What are the evidences of factors and influencing SMA?
3. What are the sources and common antibiotics used for Self-medication?
4. What are the symptoms leading/health conditions to antibiotics Self-medication?

#### Eligibility criteria

The study will use an amended PICOS (Population, Intervention, Comparison, Outcomes and Study setting) framework to determine the eligibility of the research question. The PICOS is represented in Table 1.

**Table 1:** Framework for determining eligibility of research questions (PICOS)

Criteria	Determinants
Population	Adults
Intervention	Self-medication with antibiotic
Comparison	Not applicable
Outcomes	Primary outcomes: prevalence, factors, reasons, source of antibiotics, common used antibiotics, related health conditions
Setting	Low- and middle-income countries

#### Inclusion criteria

The inclusion criteria are as follows:

❖ Will be including in the search studies that show evidence on SMA on adults;

❖ Will be including in the search studies that show evidence of intervention on SMA;

❖ Will be included in the search studies that include the following outcomes: factors influencing SMA, reasons for SMA, common used antibiotics and related health conditions;

❖ Searching will include studies done in LMICs.

#### Exclusion criteria

The exclusion criteria are as follows:

❖ Studies that where published before 2007 will be excluded from the search;

❖ Studies evidencing intervention on utilization of prescribed antibiotics.

#### Identifying relevant studies

Primary research articles, published in peer-reviewed journals, and review articles that addresses the main research question will be included in this study. All study designs will be included in this review. Databases that will be used to source literature include PubMed, CINAHL with full text, Health Source—Consumer Edition and MEDLINE; World Health Organization (WHO) and governmental websites will also be examined for policies and reports. The researchers will also use reference lists and existing networks such as organizations and conferences to source relevant literature. The search terms will include “self-medication with antibiotics”, “prevalence”, “factors influencing SMA” and “reasons”. This review will search studies that were published in any language, i.e., there will be no restriction on the language in which manuscripts were published.

### Study selection

**Fig. 1:**
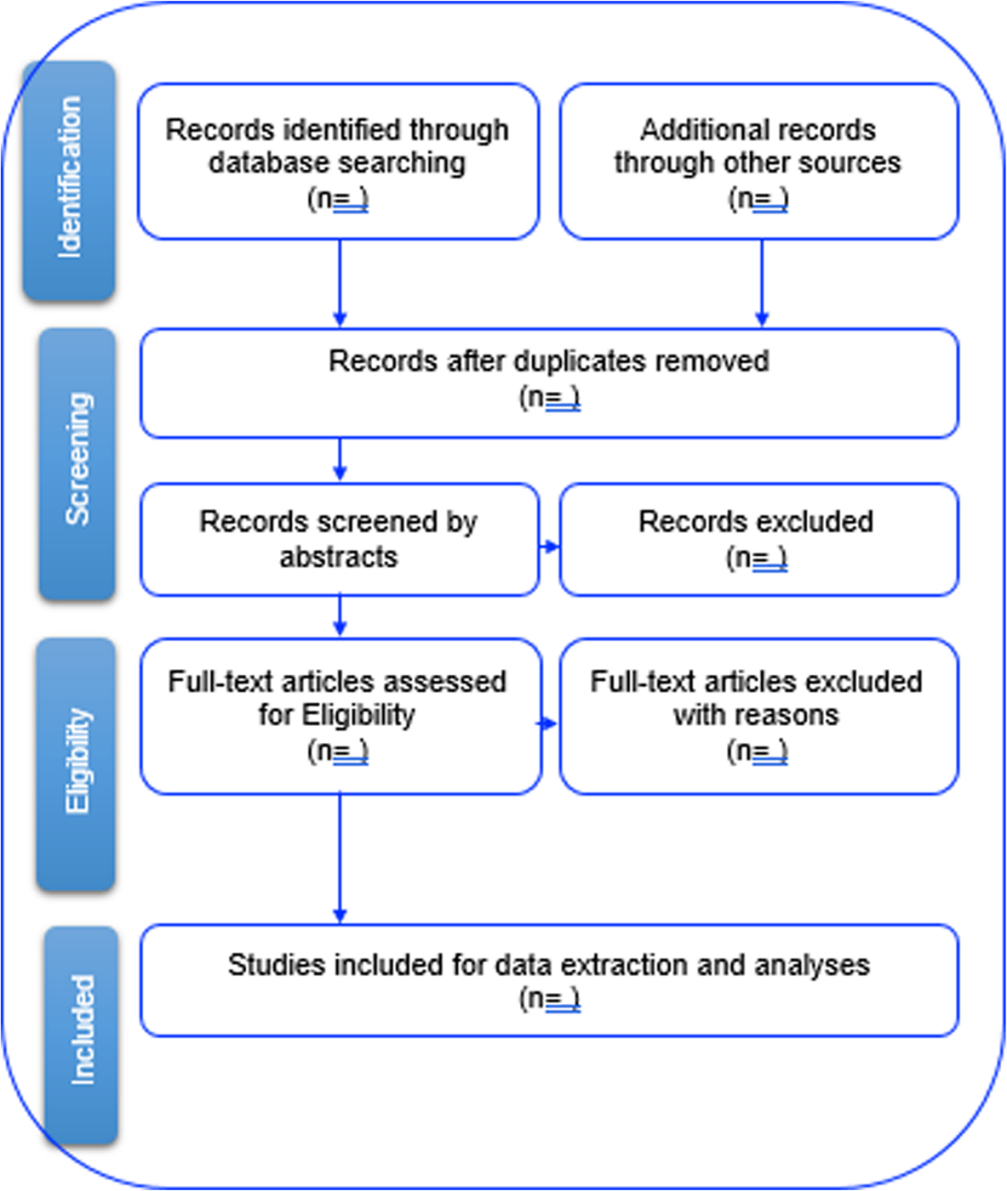
Study selection procedure

The study selection procedure will be summarized using a PRISMA flow chart as indicated in Fig. 1. The researchers will conduct a comprehensive title screening; all studies that do not address the study’s research question will be excluded along with all the duplicates. All included studies for abstract screening will be uploaded on Endnote X7 software. In the case of articles that are difficult to find, the researchers will seek assistance from the UKZN library services. Authors shall also be contacted to request the full copies of articles that are not attainable within the UKZN library database. The final Endnote database will be shared among the review team for abstract screening, i.e., two independent reviewers will screen the abstracts and full articles through guidance from the inclusion criteria. Copies of full articles for eligible studies will be obtained and maintained for data extraction.

### Charting the data

An analytical method would be utilized to extract the background information and process oriented information of each included study. Charting form will be constantly updated. A data charting form would be developed and piloted. The variables and themes included to answer the question will be determined as indicated in Table 2. The form will include bibliographic details, study setting, study design, sample size of participants, intervention, significant findings and conclusions for the primary and the secondary outcomes of the intervention.

**Table 2:** Data charting form

Author and date	
Study title	
Study design	
Aim	
Location	
Sample size	
Participant characteristics	
Intervention	
Intervention outcomes	
Most relevant findings	
Conclusions	
Comments	

### Collating, summarizing and reporting the results

The study will present a narrative account of findings from the existing body of literature through a thematic content analysis of the extracted research work. The themes will be structured around the following interned outcomes: Prevalence, factors, reasons for SMA, source of antibiotics, common used antibiotics and related health conditions. Thereafter, the themes will be coded in correspondence to the respective authors. Emerging themes will also be reported. The researchers will use a google form, word form for data extraction and NVivo version 11 for thematic analysis. The below process will be followed:

❖ Coding

❖ Categorize codes into major themes

❖ Build theme-related themes (cut-and-paste technique)

❖ Display data

❖ Identify patterns in the data and identify sub-themes

❖ Summarize

The authors will interrogate the resulting themes and critically examine their relationship to the research question. A scrutiny of the meanings of the findings as they relate to the overall aim of the study and the implications for future research shall also be carried out.

### Synthesis

The resulting themes will be analyzed and critically examine their relationship to the research question. The reviewers will also analyze the meanings of the findings in relation to the aim of the study and the implications of these findings for future research, policy and practice.

### Quality appraisal

The quality of the studies will be determined through study appraisal using the mixed method appraisal tool (MMAT)-Version 2011 [15]. The tool will be utilized to examine the appropriateness of the aim of the study, adequacy and methodology, study design, participant recruitment, data collection, data analysis, presentation of findings, authors’ discussions and conclusions. The quality of the article will be determined from the examination of the abovementioned aspects.

## Discussion

The scoping review will be conducted as a first part of a study on the factors influencing SMA in Maputo City, Mozambique. This systematic scoping review aims at mapping literature evidence on the factors and practices influencing SMA in LMCs. The purpose is to establish the extent of existing research on the dynamics of SMA in LMICs. Although studies on antibiotic use are taking place in some of these countries, there is still a scarcity of evidence on the factors that influence SMA.

The researchers will limit the search to include published studies from 2007 to 2017. A 10-year literature search is more likely to yield a comprehensive and balanced account of previous and current research in the area. Furthermore, the timeframe will allow the study to capture both the past and emerging perspectives on interventions on antibiotic use.

This review will exclude studies that report evidence on self-medication with other prescription-only drugs, as the focus is on the use of antibiotics, the access (availability, affordability), and the user’s behaviour (factors, reasons and practices). Due to how the focus of the study is on the evidence of inappropriate use of antibiotics, studies reporting evidence of individuals using prescribed antibiotics will not be included in this review. It is important for the study to incorporate factors influencing SMA in research to better understand the phenomena and design evidence-based strategies to promote good practices on antibiotic utilization.

The researchers anticipate finding relevant literature on SMA in LMICs. The acquired results will provide documented evidence on factors influencing SMA and will help identify priorities for primary research in this area. In guiding future research work, the dissemination plan for this study will include presentation for students at different levels, public health institutions, local stake holders, conference presentations and publication in journals.

## Abbreviations

LMICs: Low- and middle-income countries
MMAT: Mixed methods appraisal tool
PICOS: Population intervention outcomes study setting
SDG: Sustainable Development Goals
SMA: Self-medication of antibiotics
UKZN: University of Kwazulu Natal
WHO: World Health Organization
